# Role of sympathetic nerve activity in the process of fainting

**DOI:** 10.3389/fphys.2014.00343

**Published:** 2014-09-15

**Authors:** Satoshi Iwase, Naoki Nishimura, Tadaaki Mano

**Affiliations:** ^1^Department of Physiology, School of Medicine, Aichi Medical UniversityNagakute, Japan; ^2^Gifu University of Medical SciencesSeki, Japan

**Keywords:** syncope, vasovagal syncope, orthostatic hypotension, muscle sympathetic nerve activity, neurally mediated syncope

## Abstract

Syncope is defined as a transient loss of consciousness and postural tone, characterized by rapid onset, short duration, and spontaneous recovery, and the process of syncope progression is here described with two types of sympathetic change. Simultaneous recordings of microneurographically-recorded muscle sympathetic nerve activity (MSNA) and continuous and noninvasive blood pressure measurement has disclosed what is going on during the course of syncope progression. For vasovagal or neurally mediated syncope, three stages are identified in the course of syncope onset, oscillation, imbalance, and catastrophe phases. Vasovagal syncope is characterized by sympathoexcitation, followed by vagal overcoming via the Bezold-Jarisch reflex. Orthostatic syncope is caused by response failure or a lack of sympathetic nerve activity to the orthostatic challenge, followed by fluid shift and subsequent low cerebral perfusion. Four causes are considered for the compensatory failure that triggers orthostatic syncope: hypovolemia, increased pooling in the lower body, failure to activate sympathetic activity, and failure of vasoconstriction against sympathetic vasoconstrictive stimulation. Many pathophysiological conditions have been described from the perspectives of (1) exaggerated sympathoexcitation and (2) failure to activate the sympathetic nerve. We conclude that the sympathetic nervous system can control cardiovascular function, and its failure results in syncope; however, responses of the system obtained by microneurographically-recorded MSNA would determine the pathophysiology of the onset and progression of syncope, explaining the treatment effect that could be achieved by the analysis of this mechanism.

## Introduction

Syncope is defined as a transient loss of consciousness and postural tone, characterized by rapid onset, short duration, and spontaneous recovery, due to global cerebral hypoperfusion that most often results from hypotension. The accurate definition does not include the unconsciousness not caused by cerebral hypoperfusion, for example, epileptic seizures, concussion, and cerebrovascular accidents, which are persistent structural impairments, and persistent states of unconsciousness, for example, coma and cerebrovascular diseases (Ross, 1988).

The pronunciation of “syncope” can differ from person to person; however, [síηkəpi] seems to be generally accepted, meaning “strike” or “cut-off” in ancient Greek.

The human autonomic nervous system regulates the systemic arterial pressure to maintain constant cerebral perfusion under conditions of uneven fluid shift caused by postural change. Syncope is the state of failure of this regulating function.

Many forms of syncope are preceded by a prodromal state that often includes dizziness and loss of vision, loss of hearing, loss of pain and feeling, nausea and abdominal discomfort, weakness, cold sweating, a feeling of hotness, palpitations and other phenomena, which are often called “presyncope” (Reeves and Swenson, 2012).

In the present review, the process of syncope progression is described with two types of sympathetic change.

## Autonomic control of the cardiovascular system

Baroreceptors are situated at the carotid sinus and the aortic arch; they monitor the arterial pressure and transmit information on this pressure to the central nervous system (Mathias and Bannister, 2002). Upon extension of the arterial wall by the raising of the blood pressure, the baroreceptors (stretch receptors) generate an impulse depending on the arterial pressure via the glossopharyngeal nerve (CN IX) from the carotid sinus and via the vagus nerve (CN X) from the aortic arch, and transmit the signals to the nucleus tractus solitarius (NTS) in the medulla (Iwase, 2002, 2006, 2009).

From the NTS, excitatory information is sent via the glutamatergic neurons to the caudal ventrolateral medulla (CVLM), where inversion of positive and negative signs is executed. The CVLM then transmits GABAergic (using γ-amino butyric acid as a neurotransmitter) neurons to the rostral ventrolateral medulla (RVLM). The topographical regional differentiation has been established (Dampney, 2004).

The RVLM neurons are considered to be premotor neurons for the preganglionic efferent neurons whose cell bodies are situated in the intermediolateral nucleus (IML), with synapses to the preganglionic B-fiber neurons, and which exit from the spinal cord as white rami (Netter, 1983).

On the other hand, input from NTS to the nucleus ambiguus and the dorsal nucleus of the vagus modulates the activity of the vagal nerve, innervating the heart and presphincters of the resistant vessels, and causing decrease in heart rate and cardiac contractility (Patten, 1996).

**Figure 1:**
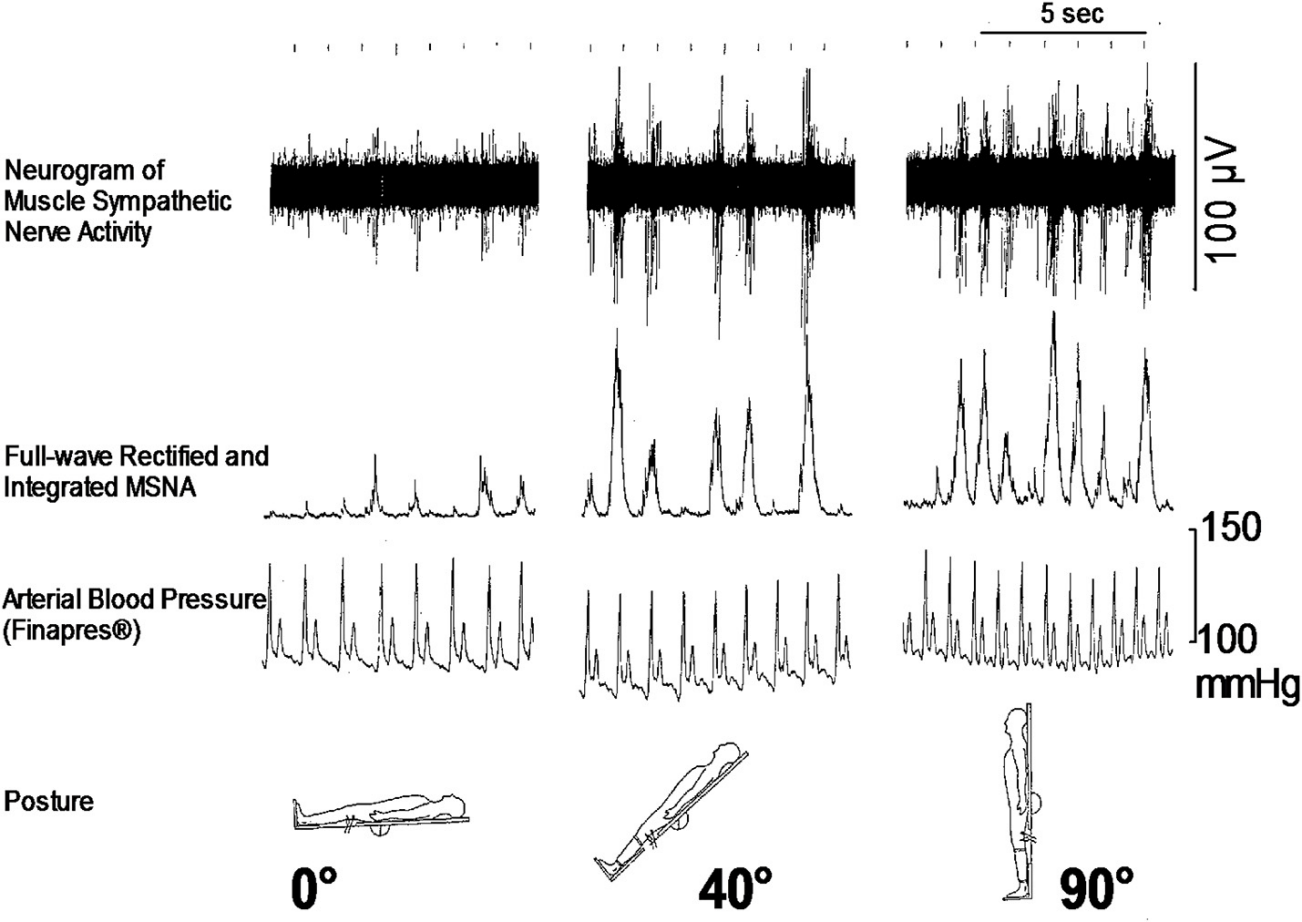
**Responses of microneurographically recorded muscle sympathetic nerve activity during orthostatic challenge**. MSNA is sparsely discharged in a supine position, but is remarkedly enhanced as the tilt angle becomes large.

As for the sympathetic efferent pathway, the cardiac sympathetic activity, and the activity of muscle sympathetic nerves innervating skeletal muscles play a role in blood pressure regulation. Muscle sympathetic nerve activity (MSNA) can be recorded microneurographically from human peripheral nerves *in situ*, which is the only baromodulatory sympathetic nerve activity directly recordable in humans (Figure 1). The techniques to record MSNA are described elsewhere (Mano et al., 2006).

These pathways provide a negative feedback mechanism to suppress the sympathetic nerve activity while the vagus nerve activity is activated when blood pressure rises, and facilitate the sympathetic nerve activity and suppress the vagus nerve activity when blood pressure drops.

## The autonomic nervous system and syncope

How is the autonomic nervous system involved in the onset of syncope? It is primarily responsible for sympathetic responses toward environmental challenges. The most frequent challenge triggering syncope might be a postural change (Figure 1). Syncope or fainting associated with orthostatic challenge includes vasovagal syncope (or in a wider sense, neurally mediated syncope) and orthostatic syncope (or orthostatic intolerance). The symptoms of these two situations seem to be the same, but the pathophysiology is different; they involve opposite directions of pathways of the autonomic nervous system.

In the following sections, syncope from the perspectives of the autonomic nervous system is described, based on the premise that no structural or conduction disorder is present in the heart.

## Vasovagal syncope (or in a wider sense, neurally mediated syncope)

The concept of vasovagal syncope was first proposed by Lewis in 1932, with primary symptoms of hypotension and bradycardia. Vasovagal syncope is triggered by an exaggerated sympathetic response to a situation; in other words, sympathoexcitation precedes the onset.

The Bezold-Jarisch reflex plays an important role in the onset of this type of syncope. This reflex is generated by the cerebral hypoperfusion due to vagal-activation-mediated sympathosuppression for the protection of myocardium (Wallin and Sundlöf, 1982). This vagal activation may be caused by hyperactivity of the left ventricular wall, which activates the stretch receptors in the wall and trabeculae, and in turn the C-fiber afferents to NTS, which triggers bradycardia and decreased myocardial contractility (Salo et al., 2007).

Several factors are known to exacerbate and accelerate the vasovagal syncope including (1) fatigue, dehydration, and hypovolemia due to hemorrhage, followed by reduced venous return, (2) blood shift and pooling in the lower body, (3) hypersensitivity of the stretch receptors in the left ventricular wall, and (4) fear, emotional stress, and reaction to pain (Fenton et al., 2000). Among these, (3) is the trigger that is considered to be essential for the Bezold-Jarisch reflex.

**Figure 2:**
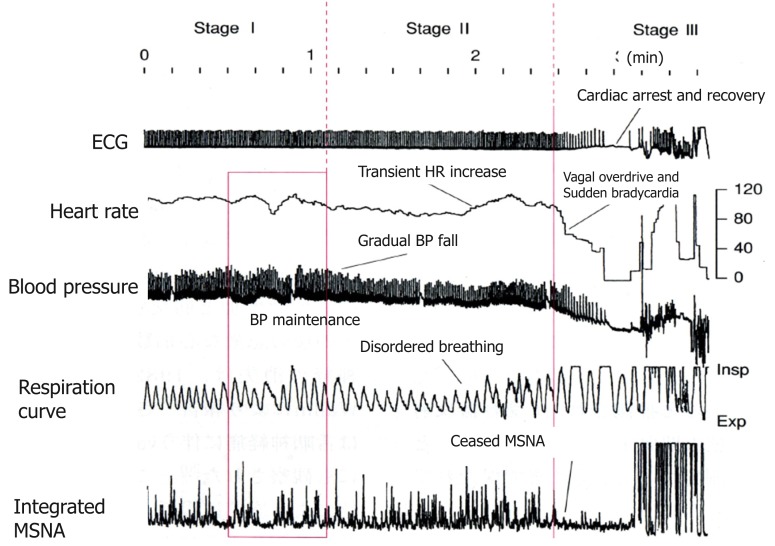
**Polygraph of vasovagal syncope**. Over the course of syncope progression in three stages, syncope onset develops at the stage III.

**Figure 3:**
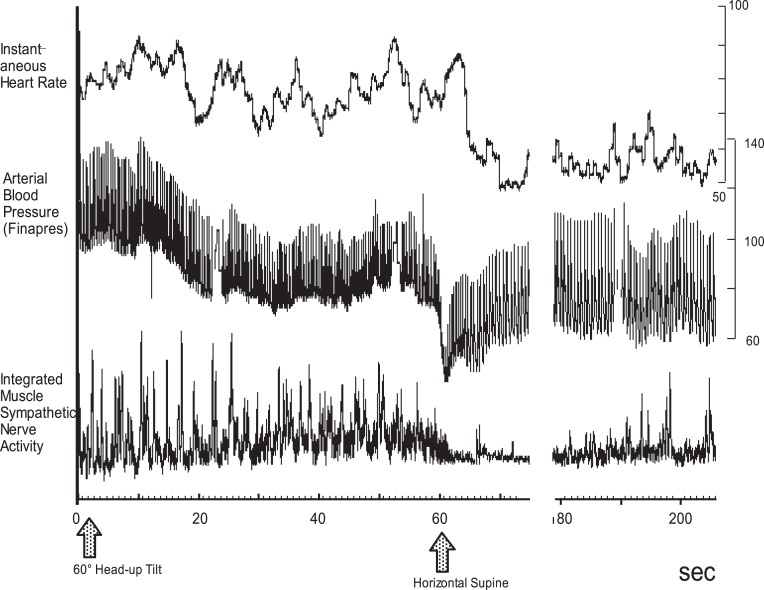
**Progression of vasovagal syncope from stage I to III**.

An analysis of the polygraph of the vasovagal syncope indicated that there are three phases in the onset of vasovagal syncope. The changes during 60° head-up tilt and polygraph trace (Figure 2) and flow charts are described below (Figure 3).

1. Stage I: oscillation phase (Sugiyama et al., 1996; Iwase, 2002, 2009; Schwartz et al., 2013)
  In this stage I, the sympathetic nerve activity (MSNA) and blood pressure display an oscillation pattern with the levels been well maintained. The time lag from MSNA discharge to blood pressure rise by the constriction of arterioles is approximately 4–5 s, while the time lag from the blood pressure rise to the vasodilatation by the suppression of sympathetic nerve activity is approximately 1 s from the descending pathway in the medulla oblongata to the peripheral vasodilating sphincter muscles via the synapse in the sympathetic chain to the postganglionic C-fiber; the time delay from the baroreceptor to the recording site of microneurography. The sympathetic discharge induces a blood pressure rise. This double feedback mechanism oscillates to maintain the blood pressure. In stage I, blood pressure wave and heart rate oscillate with a frequency of 0.1 Hz (10 s period), and the low frequency component (LF) of heart rate variability is enhanced. This phase includes mutual increases in both sympathetic and vagal activities.
2. Stage II: imbalance phase (Iwase, 2002, 2009; Kamiya et al., 2005; Schwartz et al., 2013)
  At this stage, venous return is reduced due to the failure to compensate for the enhanced venous pooling, which makes blood pressure maintenance difficult. The tidal volume is raised to increase the venous return. Failure of the double feedback mechanism arises with the enhanced power of the high frequency component (HF) of the blood pressure wave and heart rate variability. The falling blood pressure due to decreased stroke volume cannot be compensated for by only the peripheral vasoconstriction, inducing an extremely elevated heart rate. This tachycardia compensates for the reduced stroke volume, which could not be compensated for by decreased venous return or circulatory blood volume. In this state, cardiac echography sometimes reveals parallel movement of the interventricular septum and the left ventricular wall, which is called “paradoxical movement.” It is estimated that this state involves the sympathetic nerve activity being overwhelmed by the parasympathetic nerve activity, and the reciprocal relationship between the diastolic blood pressure and MSNA, denoting the baroreflex function, disappears late in this phase.
3. Stage III: catastrophe phase (Iwase, 2002, 2009; Iwase et al., 2002)
  In spite of the effort to maintain the blood pressure by an increased heart rate, the stage transitions to stage III, when a sudden heart rate drop with the cessation of MSNA is observed, resulting in an immediate fall in blood pressure. Some patients experience cardiac arrest simultaneously with cold sweat, nausea, blackout, and loss of consciousness. Dominant activation of parasympathetic nerve activity occurs concomitantly with sympathosuppression. In “neurally mediated syncope,” this stage corresponds to the classification of cardiosuppressive, vasosuppresive, or mixed types, which are based on whether the suppressive drive is dominant over cardiac or vasomotor sympathetic drives or both.

Therefore, sympathoactivation is observed, followed by sudden tachycardia, which triggers the onset of vasovagal syncope or neurally mediated syncope.

## Orthostatic hypotension or orthostatic intolerance

The orthostatic hypotension or orthostatic syncope, or orthostatic intolerance associated with postural change is caused by the response failure or lack of sympathetic nerve activity to an orthostatic challenge. A change in posture from a supine to an upright position in humans induces a fluid shift of 300–800 mL from the thoracic cavity to the lower body, including abdominal cavity or legs, resulting in a reduction in venous return. This causes a stroke volume decrease, which in turn activates the sympathetic nervous system via the arterial baroreflex and cardiopulmonary reflex. This sympathoactivation induces increases in heart rate, cardiac contractility, and peripheral resistance, while arterial blood pressure is maintained and cerebral blood flow is maintained constant.

In the case of activation failure, hypotension develops just after the postural change, sometimes progressing to fainting or syncope. The essence of orthostatic hypotension is failure of the compensation of the fluid shift that follows postural change. The largest difference from the neurally mediated syncope or vasovagal syncope is the latency of syncope onset after the orthostatic challenge. Usually, neurally mediated syncope or vasovagal syncope is induced when the individuals maintain an upright position for a comparatively long duration, possibly >10 min, whereas orthostatic hypotension develops in a short period. In tilt studies on 66 subjects, the onset latency was reported to be >1 min for 58 and >2 min for the remaining 8 individuals (Gehrking et al., 2005). One more large difference from neurally mediated or vasovagal syncope is that there are no prodromal symptoms before the onset, namely, vagal symptoms, including cold sweating or nausea. Therefore, syncope without vagal symptoms usually progresses with onset 2 min after a change of posture.

There are four causes of compensatory failure: (1) hypovolemia, (2) increased pooling in the lower body, (3) failure to activate the sympathetic activity, and (4) failure of vasoconstriction upon sympathetic vasoconstrictive stimulation.

**Figure 4:**
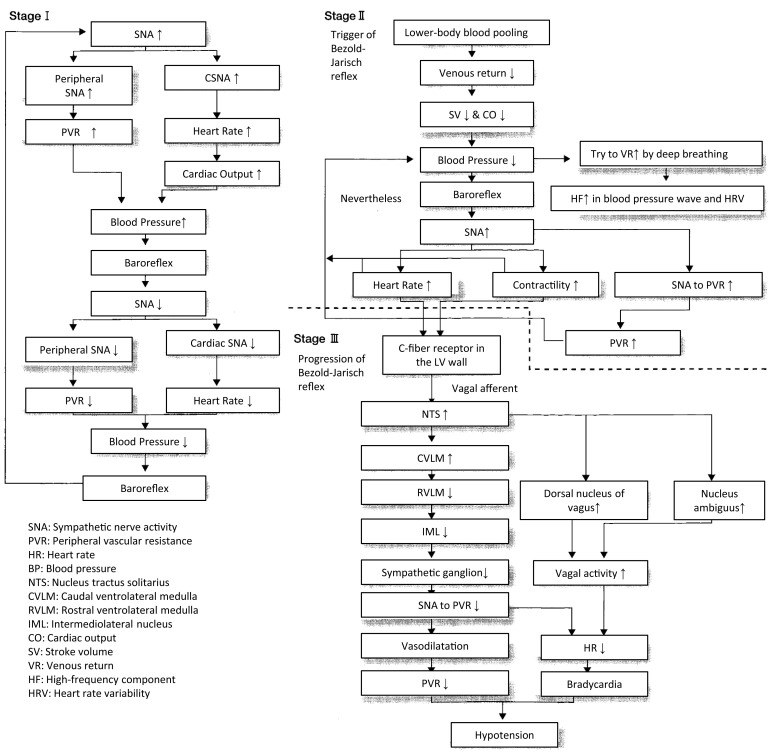
**Progression of orthostatic hypotension after 3 days of simulated microgravity exposure, dry immersion**. MSNA was gradually decreased, and blood pressure drop was also observed progressively. At 60 s after the tilt, gradually blood pressure fell, and MSNA was suppressed, while heart rate was elevated.

1. *Hypovolemia*. Hypovolemia is the most prevalent cause of orthostatic hypotension, having associations with a hot environment, dehydration, hemorrhage, diarrhea, Addison's disease, and administration of diuretics. Hypovolemia results in a fluid shift to the lower body, causing insufficient venous return, stroke volume, and cardiac output, which lowers cerebral perfusion and induces syncope. A large fluid shift increases the heart rate, but usually insufficiently. If the increase is exaggerated, this might be a cause of neurally mediated or vasovagal syncope. In this case, the blood pressure might be maintained for several minutes, while gradual blood pressure decrease might be a cause. Here, the difference between neurally mediated or vasovagal syncope and orthostatic hypotension might be reduced responsiveness of the sympathetic nerve activity to fluid shift.
2. *Increased pooling in the lower body*. This occurs due to (3) or (4) below, or due to the increased pooling in the lower body in pathological conditions including vascular disease that reduces the elasticity of the vessels, as seen in varices or pregnancy. The mechanism of development is the same as in (1).
3. *Failure to activate the sympathetic activity*. Any disorder that impairs the baroreflex function is included in this category, including carotid deafferentation, carotid sinus neuropathy, primary autonomic failure (Shy-Drager type of multiple system atrophy, MSA-P), pure autonomic failure, Parkinson's disease with autonomic failure, and acute pandysautonomia. In addition to the primary autonomic failures, secondary ones include diabetic neuropathy, amyloid neuropathy, chronic renal failure, alcoholic neuropathy, spinal cord injury, brain tumors, and postoperative symptoms of lumbar sympathectomy for the treatment of Buerger's disease or Raynaud's syndrome all have an impaired efferent sympathetic pathway, which induces marked pooling of the body fluid (Hasegawa and Koike, 2004). One more state that causes orthostatic hypotension is depression. Structural brain abnormalities are associated with depression in the elderly, suggesting a link between vascular diseases to white matter atrophy. Gordon et al. (2011) reported the association between the degree of orthostatic hypotension and white matter hyperintensity volume in the depression group indicating that systolic blood pressure drops may be a factor contributing to white matter lesions in depression in the elderly.
4. *Attenuation of vasoconstrictor response to sympathetic stimulation*. Resistant vessels constract to enhance peripheral resistance, resulting in a rise in arterial blood pressure, while lack or attenuation of the vasoconstrictor response to sympathetic stimulation might be the cause of orthostatic hypotension. This attenuated response is primarily caused by the administration of vasodilative agents including α-adrenoreceptor blockers (e.g., prazosin), calcium antagonists (nifedipine), prostaglandin E_1_ (limaprost), or nitroglycerin. We previously reported that although the microneurographically recorded sympathetic nerve activity is not altered, the effectiveness of the sympathetic activity on blood flow reduction is attenuated (Iwase et al., 2000). Orthostatic intolerance that is sometimes observed after long exposure to microgravity or a long period of bed rest is partly caused by this attenuated vasoconstrictive response to sympathoexcitation. In this case, the heart rate is not usually reduced or is in fact elevated by blood pressure reduction although MSNA gradually ceases (Figure 4).

## Sympathetic nerve activity recording during syncope

Several examinations have been proposed to investigate the pathophysiology of syncope, among which we propose recording the MSNA from the tibial nerve microneurographically simultaneously with ECG, non-invasive continuous blood pressure wave (Finapres, Finomonitor, Jentow, etc.), and fluid shift measurements by the bioimpedance method. Several investigations related to syncope with polygraphic recording have been reported by the recording of microneurographically recorded MSNA, among which Kamiya et al. reported the disappearance of 10 s oscillation in MSNA and blood pressure in tilt-induced syncope (2005), Ichinose et al. reported gradual blood pressure drop with disappearance of 10 s oscillation in LBNP (lower body negative pressure)-induced syncope (2006), and Cooke et al. reported oscillation disappearance in LBNP induced syncope (2009). Vagal suppression seemed to be classified into cardiosuppressive and vasosuppressive types, including a mixed type (Brignole et al., 2001, 2004). These two types were also evidenced by the MSNA recording by Vaddadi et al. (2010) and Fu et al. (2012), which showed suppression of MSNA, sudden bradycardia, or both. Thus, these reports clarified the pathophysiology of the progress of syncope by recording microneurographic MSNA and continuous blood pressure. In other words, in cardiosuppressive type, sudden bradycardia occurs without MSNA disappearance, whereas MSNA ceases in vasosuppressive type. These two types of vasovagal syncope should be registered.

In contrast to the activation-suppression process of MSNA in vasovagal syncope, the inability to follow the sympathetic nerve activity to hypotension is considered to cause the orthostatic intolerance. Clarification of the pathophysiology is difficult because MSNA is seldom recorded from patients with such neurological impairments who are likely to have orthostatic hypotension. We have recorded Shy-Drager syndrome (multiple system atrophy, autonomic failure type), and reported that although MSNA appeared not to be discharged from the tibial nerve, administration of L-*threo*-DOPS (L-*threo*-3,4-dihydroxyphenylserine), a precursor of noradrenaline, ameliorated the orthostatic intolerance and induced MSNA discharge from the tibial nerve (Kachi et al., 1988, Figure 5). The same phenomena were also observed in MSA-P with postprandial hypotension, in that the appearance of MSNA improved the symptom by the use of a nose drop of vasopressin (Hakusui et al., 1991).

**Figure 5:**
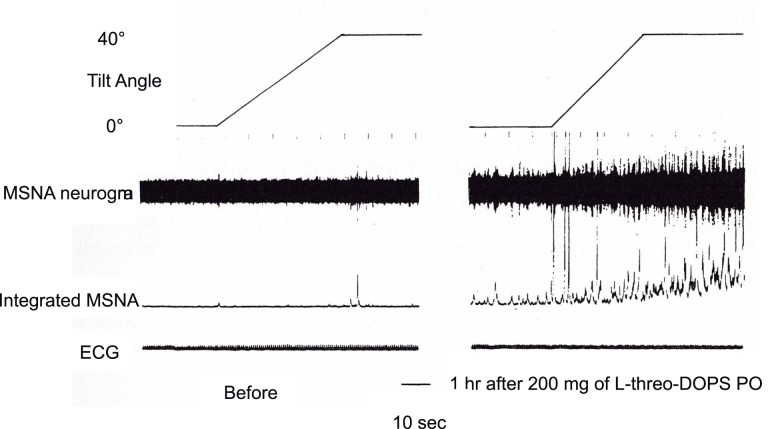
**Changes in MSNA discharges before and after administration of L-threo-3, 4-dihydroxyphenylserine (L-DOPS)**. Before the administration, there were few MSNA discharges, and enhancement due to tilting of the bed at 40° was small (left plate). The discharges became apparent 1h after L-DOPS administration and were further enhanced by 40° head-up tilting (right plate).

**Figure 6:**
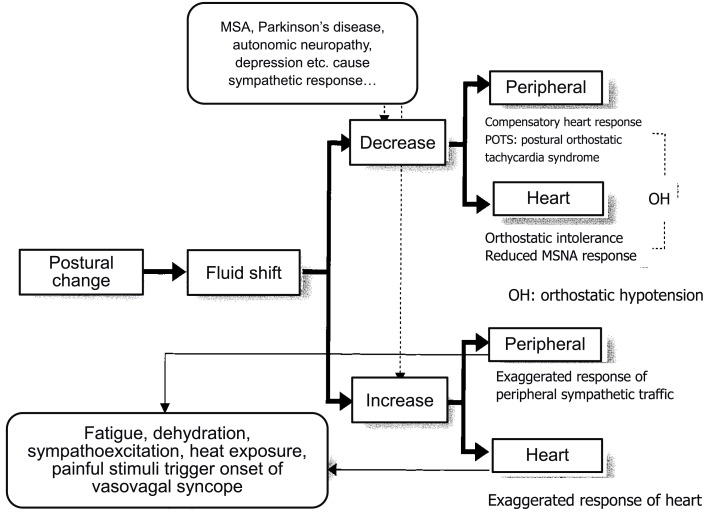
**Responses of sympathetic nerve activity at the onset of syncope**.

Thus, these changes recorded by polygraphy during the fluid shift maneuver provided measures for diagnosing orthostatic hypotension or vasovagal syncope (Figure 6). By the response of the sympathetic nervous system, we can differentially describe the underlying pathophysiology of orthostatic hypotension and vasovagal (or neutrally mediated) syncope.

## Conclusion

The sympathetic nervous system can control the cardiovascular function, and its failure results in syncope; however, responses of the system by microneurographically recorded MSNA would determine the pathophysiology of the onset and progression of syncope. An effective treatment could be achieved by the analysis of this mechanism.

### Conflict of interest statement

The authors declare that the research was conducted in the absence of any commercial or financial relationships that could be construed as a potential conflict of interest.
